# Prevalence and Severity of Oral Conditions in Elite Athletes: A Systematic Review and Meta-Analysis

**DOI:** 10.3390/dj13120589

**Published:** 2025-12-08

**Authors:** Fátima Campana Zamudio, Victor Sebastián Aleman Soto, Diego Azañedo, Akram Hernández-Vásquez

**Affiliations:** 1 Facultad de Ciencias de la Salud, Universidad Científica del Sur, Lima 15842, Peru; 100040880@cientifica.edu.pe (F.C.Z.); 100043467@cientifica.edu.pe (V.S.A.S.); dazanedo@cientifica.edu.pe (D.A.); 2 Centro de Excelencia en Investigaciones Económicas y Sociales en Salud, Vicerrectorado de Investigación, Universidad San Ignacio de Loyola, Lima 15024, Peru

**Keywords:** oral health, athletes, dental caries, periodontal disease, meta-analysis

## Abstract

**Background:** Oral health problems are common among elite athletes, yet the evidence remains fragmented and inconsistent. **Objectives:** To estimate the prevalence and severity of oral conditions in elite athletes through a systematic review and meta-analysis. **Methods:** Studies published in English, Spanish, or Portuguese, with observational design, available in PubMed, Embase, CINAHL, Web of Science, Scopus, Dentistry & Oral Science Source, and LILACS databases until 5 January 2025, were included. A narrative synthesis was used to describe the studies, and a meta-analysis of prevalences was performed using a random-effects model. Study quality assessment was performed using the Joanna Briggs Institute Critical Appraisal tools. **Results:** A total of 10 articles were included in the systematic review and meta-analysis. The overall combined prevalence of caries was found to be 44.4% (95%CI: 33.9–55.1%), the prevalence of dental erosion was 36.5% (95%CI: 22.6–51.7%), the prevalence of gingivitis was 41.4% (95%CI: 14.7–71%), the prevalence of pericoronitis was 18.7% (95%CI: 2.3–45.4%), the prevalence of periodontitis was 10.8% (95%CI: 2.7–23.3%) and the prevalence of orofacial trauma was 15.6% (95%CI: 5.3–29.7%). High heterogeneity was observed across studies. Most studies presented limitations related to participant recruitment and sample size adequacy. **Conclusions:** Dental caries, gingivitis, and dental erosion are highly prevalent among elite athletes, underscoring the importance of integrating oral health assessments into sports medicine care. High heterogeneity across studies limits the precision of prevalence estimates, emphasizing the need for standardized methodologies in future research.

## 1. Introduction

Oral conditions are key indicators of general health and well-being [1], as the oral cavity acts as an entry point for food and microorganisms [2]. Oral pathologies represent a significant public health problem, affecting more than 3.5 billion people worldwide [3,4]. With a prevalence of 45% in 2022, these conditions are among the most common non-communicable diseases (NCDs) [5]. It is estimated that the Southeast Asian and Western Pacific regions present the highest global burden of oral pathologies due to their high population density [5]. Additionally, the associated annual costs, both direct and indirect, amount to 387 billion and 323 billion dollars, respectively, reflecting the substantial economic impact of these diseases [5].

Patients with oral pathologies face difficulties in performing essential functions, as these conditions can reduce appetite, hinder food intake, and consequently lead to inadequate nutrition [6]. In particular, diseases such as periodontitis and dental caries often cause pain, sleep problems, and a decrease in the ability to perform daily tasks [7]. In elite athletes, various factors influence the development of oral pathologies, such as diet, consumption of sports drinks and nutritional supplements, use of mouthguards, training time, exposure to chemicals, dehydration, and oral dryness during exercise [8,9]. According to the literature, these athletes experience changes in mouth pH, salivary flow, microbial load, and salivary immunoglobulin A levels, in addition to presenting inadequate oral hygiene habits [10]. Therefore, it is essential that they receive regular monitoring to prevent the appearance of oral pathologies and ensure optimal oral health, which in turn can positively influence optimal sports performance.

The most common oral pathologies in elite athletes include dental trauma, caries, pericoronitis and gingivitis, among others [10,11]. In a cross-sectional study conducted with athletes participating in the London 2012 Olympic Games, it was reported that 55.1% had caries, 44.6% had dental erosions, 17.6% had suffered recent facial and dental trauma, 76% had gingivitis, 15% had periodontitis and 9.9% had pericoronitis [12]. Previous attempts to synthesize the evidence on oral health in athletes have provided valuable insights but also revealed important gaps. Ashley et al. conducted a systematic review that included 34 studies published between 1969 and 2013, reporting a wide range of caries prevalence (15% to 75%) among athletes [13]. However, this review did not perform a meta-analysis, and its inclusion criteria allowed heterogeneous study designs, including self-reports and retrospective analyses of medical records, which may have introduced considerable variability in the estimates.

Therefore, the aim of this study was to perform a systematic review with meta-analysis of the available biomedical literature on the prevalence and severity of oral conditions in elite athletes. The aim is to compile, analyze and synthesize the existing information to fill the knowledge gap, provide a comprehensive view of the subject and establish a basis for future interventions to improve oral health in this population.

## 2. Materials and Methods

### 2.1. Study Design and Registration

This systematic review and meta-analysis was reported according to the PRISMA (Preferred Reporting Items for Systematic Reviews and Meta-Analyses) statement [14] and the MOOSE (Meta-Analysis Of Observational Studies in Epidemiology) guidelines [15]. The corresponding PRISMA checklist is presented in the Supplementary File (Supplementary Material File S1. PRISMA checklist). The study protocol was registered in PROSPERO, from the Centre for Reviews and Dissemination at the University of York, with registration number “CRD42023430565” and had no deviations from it.

### 2.2. Research Question Formulation

The research question was formulated using the CoCoPop framework (Condition, Context, and Population) [16] to guide the inclusion criteria and search strategy. The research question was defined as follows: What is the prevalence and severity of oral conditions in elite athletes reported in the biomedical literature?

The elements of the CoCoPop framework were as follows:

- Condition: The main variables of interest include the prevalence and severity of oral conditions (e.g., caries, periodontal disease, dental erosions, oral lesions, orofacial trauma, among others). The conditions had to be diagnosed directly by dentists through clinical examination.

The prevalence of each oral condition was defined as: number of elite athletes with the oral condition of interest at a given time/number of athletes evaluated at a given time ×100. To be included in the analysis, studies had to explicitly report the numerator and denominator according to this definition or be calculable from the data reported in each study.

The severity of oral pathologies had to be evaluated using standardized instruments. For example, caries severity could be determined using the DMFT (Decayed, Missing, and Filled Teeth) scale [17]. Gingivitis severity could be measured using the Gingival Index [18], while periodontitis severity could be measured using the Periodontal Index or Probing Pocket Depth (PPD) [19].

- Context: Studies conducted in any competitive or sports setting.
- Population: Elite athletes, defined as those actively participating in national or international competitions in any sports discipline [20]. However, it should be considered that the definition of “elite athletes” lacks uniformity due to different factors, such as variability in the description of sports performance and inconsistency in the use of the term “elite” in the literature [21].

### 2.3. Eligibility Criteria

Studies published in English, Spanish, or Portuguese; with observational design (cross-sectional and cohort studies); with a sample size of at least ten participants per type of sport, team, or competitive event; and with sufficient data availability to calculate the prevalence or severity of oral conditions in elite athletes were included. For follow-up studies, those that included an initial measurement evaluating oral health were considered. Additionally, if several studies used common data, we selected the study with the largest sample size. Conversely, systematic reviews, short communications, case–control studies, clinical trials, letters to the editor, congress abstracts, comments, editorials, protocols, review articles, case reports and series were excluded. Publications that had not undergone a peer review process were also excluded. Furthermore, studies without access to the full text and those where the prevalence of oral conditions in elite athletes could not be determined or calculated, either because they reported an approximate prevalence or did not report absolute frequencies of cases and sample size, were excluded. Finally, studies whose population consisted of non-elite athletes, as well as those that included Paralympic athletes, were excluded.

### 2.4. Information Sources

Studies were identified through a comprehensive search of the biomedical literature available in the electronic databases PubMed, Embase, Cumulative Index to Nursing and Allied Health Literature (CINAHL), Web of Science, Scopus, Dentistry & Oral Science Source, and LILACS (Latin American and Caribbean Health Sciences Literature) until 5 January 2025. The Embase search was conducted through the Ovid interface, while CINAHL and Dentistry & Oral Sciences Source searches were conducted through EBSCO. Additionally, references of all included studies were examined to identify additional relevant studies. The systematic review was based solely on searches in previously described databases, without including the grey literature, as this does not undergo a peer review process.

### 2.5. Search Strategy

An initial search strategy was designed in PubMed to identify relevant controlled terms (MeSH Medical Subject Headings) and free terms that were combined with the Boolean operators “AND” and “OR”. Search terms were related to oral health, oral pathologies, and elite athletes. Subsequently, the search strategy was modified to adapt it to each electronic database. The search strategies were designed by a librarian specialized in biomedical systematic reviews. The initial search strategy was reviewed and validated by all study authors, including a dentist expert in systematic reviews. No restrictions were applied regarding language, publication date, publication status, publication type, or study context.

EndNote 20 was used to create a library where records identified in electronic databases were combined and duplicates were eliminated following the process described by Bramer et al. [22].

Complete details of the searches conducted can be found in the Supplementary Material Table S1.

### 2.6. Screening and Study Selection

After removing duplicates, records were imported to the Rayyan platform [23], where titles and abstracts were reviewed and classified as included or excluded according to the inclusion/exclusion criteria. Subsequently, full-text documents of included records were evaluated to define their inclusion in the study. For full-text studies that were excluded, reasons for exclusion were recorded (see Supplementary Material Table S2). These processes were conducted independently and blindly by two investigators (FCZ and VSAS), and disagreements were resolved through discussion and consensus. If disagreement persisted, a third author (AHV) participated in its resolution.

### 2.7. Data Extraction Process

Two investigators (FCZ and VSAS) independently and in duplicate extracted relevant data using a data extraction template designed in Microsoft Excel. A pilot test was conducted with three studies to ensure correct use of the template. Upon completion of data extraction, a cross-check was performed to verify concordance. Disagreements were resolved through discussion and consensus with the participation of a third investigator (AHV).

General data were extracted from each study, including study title, DOI, author(s), publication year, journal name, study location (country), sporting event where the study was conducted, study date or data collection period, study type, sport type, total participants, sex, age or age groups, diagnosed oral diseases, and number of athletes affected by each pathology. If any clinical or epidemiological data were unclear, it was discussed among all authors.

### 2.8. Methodological Quality Assessment

The Joanna Briggs Institute’s Critical Appraisal Tools for prevalence studies [16] was used, which contains nine evaluation criteria. These criteria cover the appropriateness of the sampling frame, suitability of participant recruitment, adequate sample size, detailed description of subjects and study context, data analysis based on an adequate sample, validity of measurement methods, reliability and standardization of measurement methods, appropriateness of statistical analysis, and response rate or its handling. The methodological quality assessment of included studies was conducted independently and blindly by two investigators (FCZ and VSAS). Disagreements were resolved through the participation of a third study author (AHV).

### 2.9. Data Synthesis

A narrative synthesis was performed to summarize and describe the characteristics, context, quality, and findings of each study included in the systematic review. Quantitative synthesis was performed through a meta-analysis of prevalences [24]. The pooled prevalence was calculated using a random-effects model due to significant heterogeneity between studies. Results are presented as the combined prevalence with 95% confidence intervals (95% CI). Additionally, pooled prevalences were calculated using Freeman–Tukey double arcsine transformation to stabilize variances and normalize the distribution of prevalences [24]. The between-study variance estimation used the Der Simonian–Laird method, with confidence intervals by the Clopper–Pearson exact binomial method. Both results syntheses are presented in tables and forest plots according to the oral condition.

Heterogeneity between studies was assessed using the *I*^2^ statistic, with values above 50% interpreted as moderate to high heterogeneity. Additionally, the Tau^2^ statistic was calculated to quantify between-study variance. Statistical significance of heterogeneity was assessed with Cochran’s Q test, and when the *p*-value was <0.05, the result was considered to have significant heterogeneity, and the random-effects model was used for analysis. The fixed-effects model was performed and presented for comparative purposes.

Publication bias was not evaluated, considering that, in meta-analyses of prevalence studies, funnel plots were found to be inaccurate for assessing publication bias [25].

All statistical analyses were performed in R (version 4.2.3; R Development Core Team) with the meta package for meta-analysis [26], with a *p*-value less than 0.05 indicating statistically significant differences.

### 2.10. Ethical Considerations

Institutional ethics committee approval was not requested for this study as it is a systematic review based on bibliographic data.

## 3. Results

**Figure 1:**
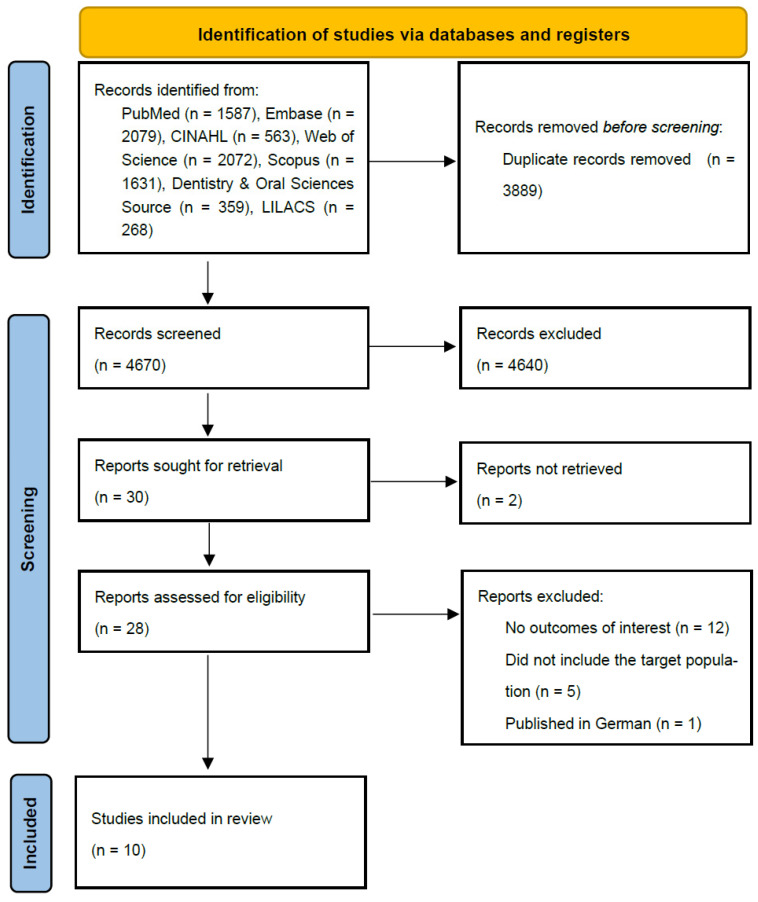
PRISMA 2020 flow diagram of study selection.

The search was conducted in June 2023 and updated in January 2025. After evaluating titles and abstracts of 4670 articles, 30 were selected for full-text review (Figure 1). Full text was not accessible for two studies [27,28]. Finally, 10 studies met the selection criteria and were included in the systematic review [11,12,29,30,31,32,33,34,35,36].

These studies were published between 2010 and 2022 and in English (Table 1). Table 1 provides an overview of the 10 included studies. Of these, seven were conducted in Europe [11,12,30,31,32,33,34], two in the Americas [29,35] and one in Asia [36]. Regarding study design, all 10 included studies had a cross-sectional design and covered a wide variety of sports. The total number of athletes evaluated was 1747, with a minimum of 17 and a maximum of 409 participants in the studies. Regarding data collection location, four studies gathered information during high-level international competitions, such as the XV Pan American Games in Rio de Janeiro (2007) [29], the London Olympics (2012) [12], the Rio de Janeiro Olympics and Paralympics (2016) [32] and the Lima Pan American Games (2019) [35]. The remaining six studies obtained data from athletes in national competitions or professional teams, including FC Barcelona Club in Spain [11], professional football players from eight UK clubs [30], multi-sport discipline clubs in the United Kingdom [31], an elite swimmers club in Portugal [33], an elite swimmers club in Portugal [34] and an elite athletes club in Peshawar, Pakistan [36].

Table 2 provides the results reported in the 10 included studies, organized by oral pathologies, and indicating their prevalence. High heterogeneity was observed among the analyzed studies for all evaluated pathologies, with *I*^2^ values ranging between 90% and 97% (*p* < 0.01). This heterogeneity justified the use of a random-effects model to calculate the combined global prevalences for caries, dental erosion, gingivitis, pericoronitis, periodontitis, and orofacial trauma.

### 3.1. Caries

**Figure 2:**
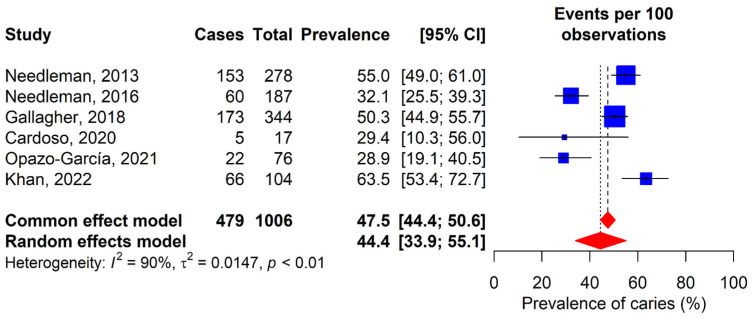
Forest plot for the meta-analysis of dental caries prevalence. 95% CI: 95% confidence interval; I^2^: percentage of heterogeneity; τ^2^: between-study variance; *p*: *p*-value. Blue squares represent individual study estimates, with the center indicating the point estimate of prevalence and the size proportional to study weight. Horizontal lines indicate 95% confidence intervals. Red diamonds show pooled estimates from common effect (upper) and random effects (lower) models, with the center representing the pooled estimate and the width indicating the 95% confidence Interval. Vertical dashed lines represent the pooled estimates from each model [12,30,31,33,35,36].

Caries prevalence was reported in six of the included studies [12,30,31,33,35,36], showing considerable variability (Figure 2). The lowest prevalence was 28.9%, observed in the study by Opazo-García et al. [35], while the highest reached 63.5%, according to Khan et al.’s study [36]. After analysis, a combined global prevalence of 44.4% (95% CI: 33.9–55.1%) was determined. Regarding severity, Gay-Escoda et al.’s study reported a DMFT score of 5.7 [11], Kragt et al. DMFT score of 3 [32], Khan et al.’s study reported a DMFT score of 2.7 [36], and De la Parte et al.’s study reported a DMFT score of 8.12 in individual sports athletes and a score of 6.1 in team sports athletes [34].

### 3.2. Dental Erosion

**Figure 3:**
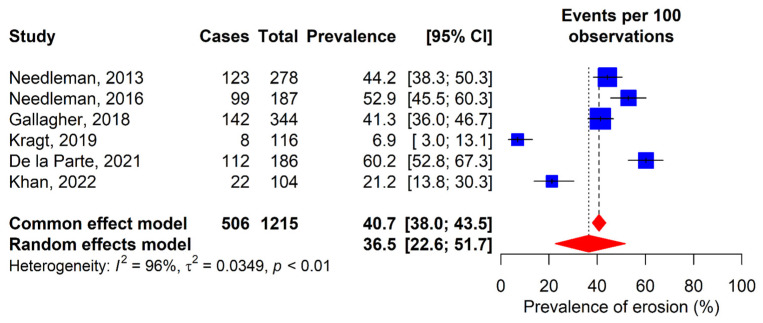
Forest plot for the meta-analysis of dental erosion prevalence. 95% CI: 95% confidence interval; I^2^: percentage of heterogeneity; τ^2^: between-study variance; *p*: *p*-value. Blue squares represent individual study estimates, with the center indicating the point estimate of prevalence and the size proportional to study weight. Horizontal lines indicate 95% confidence intervals. Red diamonds show pooled estimates from common effect (upper) and random effects (lower) models, with the center rep-resenting the pooled estimate and the width indicating the 95% confidence Interval. Vertical dashed lines rep-resent the pooled estimates from each model [12,30,31,32,34,36].

Dental erosion was evaluated in 60% of the included studies [12,30,31,32,34,36]. Prevalences range from 6.9% in Kragt’s study [32] to 60.2% in De la Parte’s study [34]. According to the random-effects model, a combined global prevalence of dental erosion of 36.5% (95% CI: 22.6–51.7%) was determined (Figure 3).

### 3.3. Gingivitis

**Figure 4:**
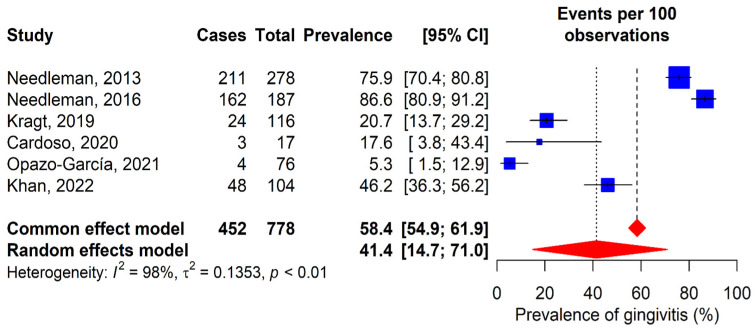
Forest plot for the meta-analysis of gingivitis prevalence. 95% CI: 95% confidence interval; I^2^: percentage of heterogeneity; τ^2^: between-study variance; *p*: *p*-value. Blue squares represent individual study estimates, with the center indicating the point estimate of prevalence and the size proportional to study weight. Horizontal lines indicate 95% confidence intervals. Red diamonds show pooled estimates from common effect (upper) and random effects (lower) models, with the center rep-resenting the pooled estimate and the width indicating the 95% confidence Interval. Vertical dashed lines rep-resent the pooled estimates from each model [12,30,32,33,35,36].

A total of six studies evaluated the prevalence of gingivitis (Figure 4) [12,30,32,33,35,36]. The observed prevalences varied from 5.3% in Opazo-García’s study [35] to 86.6% in Needleman’s study [30]. The prevalence obtained with the random-effects model was 41.4% (95% CI: 14.7–71%). Additionally, Gay-Escoda et al.’s study reported a GI score of 1.1 (SD 0.8) [11].

### 3.4. Pericoronitis

**Figure 5:**
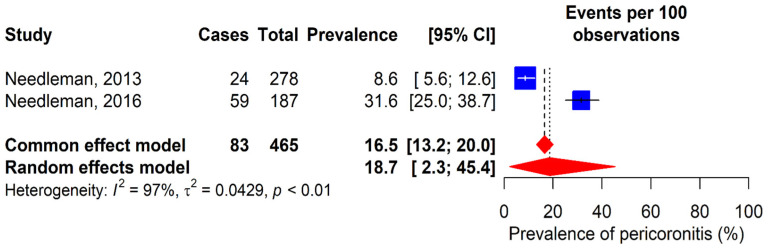
Forest plot for the meta-analysis of pericoronitis prevalence. 95% CI: 95% confidence interval; I^2^: percentage of heterogeneity; τ^2^: between-study variance; *p*: *p*-value. Blue squares represent individual study estimates, with the center indicating the point estimate of prevalence and the size proportional to study weight. Horizontal lines indicate 95% confidence intervals. Red diamonds show pooled estimates from common effect (upper) and random effects (lower) models, with the center rep-resenting the pooled estimate and the width indicating the 95% confidence Interval. Vertical dashed lines rep-resent the pooled estimates from each model [12,30].

The prevalence of pericoronitis was evaluated in two studies (Figure 5) [12,30]. Results showed variations, with 8.6% in Needleman’s study [12] and 31.6% in Needleman’s study [30]. The combined global prevalence of pericoronitis was 18.7% (95% CI: 2.3–45.4%).

### 3.5. Periodontitis

**Figure 6:**
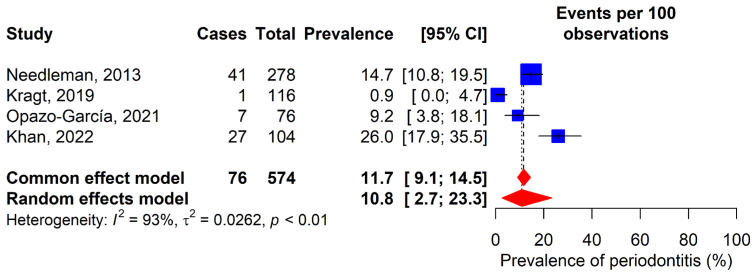
Forest plot for the meta-analysis of periodontitis prevalence. 95% CI: 95% confidence interval; I^2^: percentage of heterogeneity; τ^2^: between-study variance; *p*: *p*-value. Blue squares represent individual study estimates, with the center indicating the point estimate of prevalence and the size proportional to study weight. Horizontal lines indicate 95% confidence intervals. Red diamonds show pooled estimates from common effect (upper) and random effects (lower) models, with the center rep-resenting the pooled estimate and the width indicating the 95% confidence Interval. Vertical dashed lines rep-resent the pooled estimates from each model [12,32,35,36].

The prevalence of periodontitis was evaluated in four included studies (Figure 6) [12,32,35,36], with ranges varying from 0.9% in Kragt’s study [32] to 26% in Khan’s study [36]. According to the random-effects model, the prevalence was 10.8% (95% CI: 2.7–23.3%). Additionally, Gay-Escoda et al.’s study reported a PPD score of 1.9 [11].

### 3.6. Orofacial Trauma

**Figure 7:**
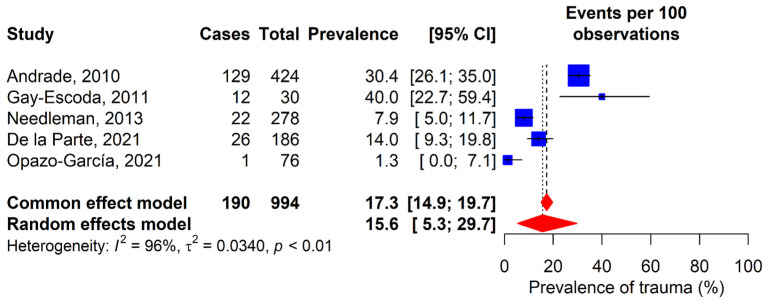
Forest plot for the meta-analysis of orofacial trauma prevalence. 95% CI: 95% confidence interval; I^2^: percentage of heterogeneity; τ^2^: between-study variance; *p*: *p*-value. Blue squares represent individual study estimates, with the center indicating the point estimate of prevalence and the size proportional to study weight. Horizontal lines indicate 95% confidence intervals. Red diamonds show pooled estimates from common effect (upper) and random effects (lower) models, with the center rep-resenting the pooled estimate and the width indicating the 95% confidence Interval. Vertical dashed lines rep-resent the pooled estimates from each model [11,12,29,34,35].

Five studies were included to analyze the prevalence of orofacial trauma in elite athletes (Figure 7) [11,12,29,34,35]. Results varied from 1.3% in Opazo-García’s study (2021) to 40% in Gay-Escoda’s study (2011). One of the studies focused exclusively on football players as the study population [11], while the remaining four included participants from various sports disciplines [12,29,34,35]. The combined global prevalence of orofacial trauma was 15.6% (95% CI: 5.3–29.7%).

### 3.7. Quality of Included Studies

Regarding the quality assessment of studies using the JBI critical appraisal tool for prevalence studies, it was observed that all 10 included studies received a negative response (No) for items 2 and 3, which refer to participant recruitment and adequate sample size (Supplementary Material Table S3). Finally, the studies by Opazo-García et al. [35] and Gay Escoda et al. [11], had the highest number of negative responses (“No”, “Not applicable” or “Unclear”) on the JBI critical appraisal tool items.

## 4. Discussion

The present study fulfilled its objective of conducting a systematic review of the biomedical literature on the prevalence and severity of oral conditions in elite athletes. Through the analysis of ten selected articles, conditions such as caries, gingivitis, pericoronitis, orofacial trauma, periodontitis, and dental erosion were addressed. The findings obtained show that the studied oral pathologies present a high prevalence in this population. These results highlight the need to consider oral health as an essential component in athletes’ general well-being, given its possible impact on sports performance and quality of life.

Of the six oral pathologies studied in this systematic review, dental caries was the most predominant in elite athletes, with a combined global prevalence of 44.4% (range 28.9 to 63.5%). Previous syntheses of the literature have documented considerable variability in caries prevalence among this population, with estimates ranging from 15% to 75% [13]. Such disparity likely reflects methodological heterogeneity across primary studies, as well as differences in inclusion criteria between reviews. For instance, earlier reviews incorporated 34 studies published between 1969 and 2013, while the present analysis focused on 6 studies published from 2013 onward. This could be due to Ashley et al. allowing a wide variety of study designs, including unvalidated self-reports through clinical examination, studies where oral conditions were determined by clinical visits for treatment, or retrospective analyses based on medical records or secondary databases. In contrast, this review focused exclusively on cross-sectional studies and baseline assessments of cohorts, where oral condition was measured by a dentist. On the other hand, the results obtained are similar to another systematic review, focused exclusively on the prevalence of caries in athletes in general, which included studies until April 2017 and estimated a prevalence of 46.25% [37]. The results of both studies converge on a common finding, a significant prevalence of caries in athletes, suggesting that, regardless of the level of sports competition, they seem to share a similar risk of developing this oral condition.

Despite these estimates, it is important to highlight that the combined global prevalence observed in this study is lower than what has been reported in some individual studies in the general population. A cross-sectional study conducted in Nepal between April and June 2022 [38], reported that the prevalence of dental caries was 59.42% in adults between 18–35 years. These findings, although not directly comparable due to methodological and population differences, demonstrate that although dental caries presents a high prevalence among elite athletes, this condition is even more frequent in the general population. This difference could be attributed to factors such as regular and frequent access to preventive dental care and greater awareness about dental health among athletes in this category, in contrast to the general population, where these resources may be less accessible or prioritized.

Regarding dental caries severity, studies included in this systematic review reported a variable DMFT index, classifying caries severity as low and very low [39]. The results of Gay-Escoda et al., Kragt et al., and Khan et al. showed very low severity [11,32,36], while De la Parte et al. reported low severity in both individual and team sports athletes [34]. When comparing these findings with studies of the general population, such as a cross-sectional study conducted in Spain in 2010 [40], which reported a DMFT index of 7.64 in adults aged 35–44 years, and another study conducted in Iran in 2015, with a DMFT index of 7.8 in adults aged 35–45 years [41], both classified as low severity according to WHO criteria, a similarity in caries severity levels between elite athletes and the general population was observed. These results suggest that in terms of severity, dental caries in elite athletes is comparable to the general population, suggesting that competitive level does not imply greater severity of this condition.

In this sense, although the severity of the condition is mostly classified as low or very low, the high prevalence of this condition in elite athletes underlines the need to implement effective measures for promotion and prevention of dental caries in this population, adapting the strategies of these interventions to the clinical characteristics of the pathology and specific risk factors associated with each sports discipline to which the athletes belong.

Another of the studied oral pathologies was dental erosion, which may be associated with intrinsic and extrinsic factors affecting oral health and, potentially, sports performance of elite athletes [42]. The pooled prevalence identified in this review (36.5%; range: 6.9–60.2%) falls within the lower bound of estimates reported in the literature, where figures as high as 85% have been documented in certain athletic populations [13]. These findings highlight the high susceptibility of elite athletes to this condition, probably due to different factors such as frequent consumption of acidic isotonic beverages, chronic dehydration, and alterations in salivary flow [9]. The high reported prevalence highlights the need to incorporate preventive strategies, as well as educational interventions and periodic monitoring. In summary, the significant prevalence of dental erosion in elite athletes, consistent with previous literature, emphasizes the importance of including multidisciplinary approaches in the dental management of this population to mitigate the long-term effects of a high-performance athlete’s lifestyle.

Gingivitis is one of the most frequent periodontal conditions in elite athletes. According to this meta-analysis, the global prevalence of gingivitis was 41.4% (14.7–71%), making it the second most predominant oral pathology in the study. Notably, this estimate is more conservative than those derived from individual studies, where prevalences exceeding 75% have been observed [13], who reported a prevalence of 76% based on a single study [12]. A narrative review examining oral pathologies in elite athletes similarly reported higher figures, ranging from 58% to 97% [43]. However, the lack of a systematic approach in that review and the absence of a combined prevalence estimate could explain the higher figures compared to the present analysis. Although the prevalence found in this review is lower compared to previously mentioned reports, this figure remains significant, evidencing the susceptibility of elite athletes to periodontal diseases.

Regarding gingivitis severity, only one of the included studies provided information on this aspect. This study reported that gingivitis severity in elite athletes was mild according to the GI [18]. This finding is consistent with the results of an observational study conducted in India, which evaluated an adult population (>18 years) [44], and reported a gingival index of 1.19, which also corresponds to mild inflammation. These results, although limited, suggest that gingivitis in this population might not reach alarming levels of severity, being even comparable to the levels of the general population.

Pericoronitis, an inflammation associated with partial dental eruption, represents a problem in oral health and quality of life of elite athletes [45]. We found a prevalence of 18.7%, while Ashley et al. reported prevalences ranging from 4.6 to 39% [13]. The differences in findings could be explained by discrepancies in the included studies: this systematic review analyzed two studies, while Ashley et al. included five, of which only one [12] was common to both reviews. The reason the remaining four studies were not considered was the eligibility criteria employed in each review. Despite not finding a remarkably high prevalence compared to other studied oral pathologies, early detection of pericoronal conditions remains important to avoid complications in elite athletes.

Periodontitis is a chronic inflammatory disease that not only affects oral health but can also influence physical performance, particularly in elite athletes [46]. The pooled prevalence of periodontitis (10.8%) aligns with contemporary estimates suggesting that severe periodontal involvement remains relatively uncommon in this population, with most published figures falling between 5% and 15% [14,43]. Differences across reviews may reflect variations in case definitions, as periodontitis classification has evolved considerably over the past decade. This review included studies such as Forrest et al. [27], Yang et al. [47], Randell et al. [48], and Bryant et al. [49], which were not included in the present review as they did not entirely study the target population, such as Bryant et al.’s study [49], which included both athletes and elite athletes in their total population, or Yang et al.’s study [47], which includes administrative personnel in its population. On the other hand, this meta-analysis, by applying stricter inclusion criteria, such as including only certain study designs and focusing solely on elite athletes, could have generated a more precise prevalence estimate for the population of interest.

Regarding periodontitis severity, one study included in the systematic review reported a mean PPD score of 1.9, meaning that the periodontitis found in this group was classified as non-severe [50]. Although periodontitis is not the most prevalent periodontal condition in this population, its clinical relevance extends beyond oral health. Notably, Khan et al. [36], one of the studies included in this review, demonstrated a statistically significant association between periodontal disease and self-reported impact on sports participation and performance among elite athletes (OR: 0.283; 95% CI: 0.097–0.824; *p* = 0.021). These findings provide direct evidence that periodontal conditions may negatively influence athletic performance, supporting the importance of a comprehensive approach to periodontal health in elite athletes aimed at optimizing both general well-being and sports outcomes.

Orofacial trauma represents a frequent condition among elite athletes, particularly in contact sports, with significant implications for their oral health and sports performance [51]. In the present study, a global prevalence of 15.6% was identified, with a wide range of variation in point prevalences (from 1.3 to 40%). The literature documents substantial variability in orofacial trauma prevalence, with estimates ranging from 14% to 57% depending on the sports discipline examined [13]. Contact sports such as rugby, ice hockey, and basketball consistently exhibit higher rates, underscoring the importance of sport-specific preventive strategies [51,52]. Although orofacial trauma is prevalent in elite athletes, the systematic review conducted by Tysiąc-Miśta et al. estimated a prevalence of orofacial trauma in the population of 19.48% [53], a figure that is higher than that reported in this review focused on elite athletes. This discrepancy could be explained by the frequent use of oral protection devices among athletes, which represent a key strategy to reduce the risk and severity of orofacial trauma, especially in contact sports [54]. However, comparison with the general population should be interpreted with caution due to the high heterogeneity of included studies, which presented prevalence ranges between 1.3% and 40%.

This study presents several limitations that should be considered when interpreting the results. Despite an exhaustive search being conducted in multiple databases, only 10 studies were included, mostly conducted in Europe, which could limit the representativeness of the findings, as some contexts might be insufficiently represented. All studies included were published in English. This may reflect a predominance of English-language publications in the field or potential language bias in the indexed literature, and relevant studies published in other languages may have been missed. Additionally, the lack of studies addressing certain specific oral conditions, such as orofacial trauma and dental erosion, makes it difficult to establish conclusions for some pathologies. Furthermore, the studies included do not report specific information on access to and timing of preventive dental care. Having this information would allow for better contextualization of the prevalences observed and determination of the extent to which the lack of systematic preventive programs contributes to the burden of oral diseases in this population. Not all studies evaluated the severity of oral conditions, which prevents conducting a more detailed analysis for each pathology. Furthermore, the included studies applied specific criteria defined by each author to group participants, which limited the classification of sports disciplines. For example, some studies differentiated between individual and collective sports, or between contact and non-contact sports, making it difficult to standardize variables and comparative analysis of data. There were also limitations related to the concept of “elite athletes”, given that there is no clear consensus that allows establishing a precise definition for this group [21]. Lastly, the included studies present variability in designs, evaluation methods, and diagnostic criteria for oral conditions. This methodological heterogeneity contributed to high statistical heterogeneity in the meta-analysis results (high *I*^2^), which limits the interpretation of combined prevalence estimates. From a theoretical perspective, various factors could contribute to this heterogeneity, such as contact sports carrying a higher risk of orofacial trauma, while water sports may be associated with greater dental erosion due to exposure to chemical agents such as chlorine. Moreover, this heterogeneity between studies was reflected in the wide variability of point prevalence estimators for different conditions, making it difficult to identify a precise global prevalence for the evaluated conditions.

Future studies should expand the geographic coverage of research, including underrepresented regions, to improve the representativeness of findings. Similarly, these studies should investigate poorly addressed oral conditions, such as orofacial trauma and dental erosion, and conduct detailed severity assessments with standardized indices. Additionally, homogenization of definitions and classifications is required, both for elite athletes and sports disciplines, along with consistent diagnostic protocols.

## 5. Conclusions

This review provides a quantitative synthesis of the prevalence and severity of key oral conditions in elite athletes, directly addressing the existing gap in consolidated evidence. The findings indicate that oral pathologies in elite athletes have a considerable prevalence and variable severity. These conditions could not only negatively impact athletes’ general health but may also compromise their sports performance. Despite the findings obtained, the high heterogeneity between studies and the lack of subgroup analyses, due to the limited number of available studies, make it difficult to draw precise conclusions about the prevalence of oral conditions in elite athletes. These limitations underscore the need for future research to adopt uniform clinical definitions, standardized assessment protocols, and clear reporting of the timing of oral evaluations in relation to training and competition. Additionally, expanding research to currently underrepresented regions and oral conditions will help strengthen the global evidence base. Nevertheless, the current findings provide an important basis for guiding future research and developing more effective strategies for prevention and promotion of oral health in this population.

## Figures and Tables

**Table 1:** Characteristics of included studies.

Author	Year of Publication	Country	Setting	Study Type	Number of Participants
Andrade et al. [29]	2010	Brazil	XV Pan American Games	Cross-sectional study	n = 409
Gay Escoda et al. [11]	2011	Spain	Soccer players of the Football Club Barcelona	Cross-sectional study	n = 30
Needleman et al. [12]	2013	England	London 2012 Olympic Games	Cross-sectional study	n = 278
Needleman et al. [30]	2016	England	Players of eight UK professional football clubs: Hull FC, Manchester United FC, Southampton FC, Swansea City AFC, West Ham FC, Brighton & Hove Albion, Cardiff FC, and Sheffield United FC.	Cross-sectional study	n = 187
Gallagher et al. [31]	2018	England	UK elite athletes from different sports	Cross-sectional study	n = 344
Kragt et al. [32]	2018	Netherlands	Olympic and Paralympic Games in Rio de Janeiro 2016	Cross-sectional study	n = 116
Cardoso et al. [33]	2020	Portugal	Young elite Portuguese swimmers	Cross-sectional study	n = 17
De la Parte et al. [34]	2021	Spain	Elite athletes from the community of Aragon	Cross-sectional study	n = 186
Opazo-García et al. [35]	2021	Peru	Pan American Games Lima 2019	Cross-sectional study	n = 76
Khan et al. [36]	2022	Pakistan	Elite athletes from Peshawar	Cross-sectional study	n = 104

n = sample size; UK = United Kingdom; FC = Football Club.

**Table 2:** Prevalence and severity of oral pathologies in elite athletes.

Author	Year	Sports	Dental Caries	Periodontitis	Gingivitis	Dental Erosion	Orofacial Trauma	Pericoronitis
Andrade et al. [29]	2010	Triathlon Aquatic marathon Artistic gymnastics Badminton Biking Figure skating Rhythmical gymnastics Sailing Wrestling Karate Taekwondo Rowling Shooting sports Archery Beach volleyball Rafting Basketball Synchronized swimming Diving Fencing Squash Team handball Baseball Volleyball Weightlifting Boxing Judo Water polo Swimming Field hockey Track and field	Not reported	Not reported	Not reported	Not reported	Triathlon 1/1 (100%) Wrestling 10/12 (83.3%) Boxing 14/19 (73.7%) Basketball 12/17 (70.6%) Synchronized swimming 2/3 (66.7%) Karate 3/5 (60.0%) Handball 8/14 (57.1%) Judo 10/19 (52.6%) Soccer 24/61 (39.3%) Water polo 7/20 (35%) Diving 1/3 (33.3%) Fencing 1/3 (33.3%) Field Hockey 10/32 (31.3%) Baseball 4/14 (28.6%) Taekwondo 2/7 (28.6%) Rowing 1/7 (14.3%) Shooting sports 1/7 (14.3%) Volleyball 1/17 (5.9%) Swimming 2/19 (10.5%) Weightlifting 1/17 (5.9%)	Not reported
Gay Escoda et al. [11]	2011	Soccer	DMFT: 5.7 Mean active caries: 2.2 (SD 3) Filled component: 2.9 (SD 3.1) Missing component: 0.6 (SD 1.0)		GI Score: 1.1 (SD 0.8)	Not reported	Dental trauma 12/30 (40%) Crown fracture: 7/30 (23.3%)	Not reported
Needleman et al. [12]	2013	Boxing Football Hockey Judo Shooting Swimming Track and field Volleyball Water polo Weightlifting Others	153/278 (55.1%)	41/278 (15%)	211/278 (75.9%)	Dental erosion: 123/278 (44.6%) Anterior: 37.6% (46/123) Posterior: 48% (59/123)	New Orofacial trauma: 22/278 (17.6%)	22/278 (17.6%)
Needleman et al. [30]	2016	Soccer	69/187 (36.9%)	Not reported	162/187 (80%)	Dental erosion: 99/187 (53.1%) Anterior: (20.9%) Posterior: (20.6%)	Not reported	59/187 (3.2%)
Gallagher et al. [31]	2018	Athletics track and field Cycling Gymnastics Hockey Rowing Rugby Football Sailing Swimming	173/344 (49.1%) Rugby 61.1% Football 61.5% Rowing 33.3%	Not reported	Not reported	142/344 (41.4%)	Not reported	Not reported
Kragt et al. [32]	2018	Unspecific	DMFT score: 3.0	1/116 (0.9%)	24/116 (21.6%)	8/116 (7.2%)	Not reported	Not reported
Cardoso et al. [33]	2020	Swimming	5/17 (29.41%)	Not reported	3/17 (17.64%)	Not reported	Not reported	Not reported
De la Parte et al. [34]	2021	Individual sports: fencing, tennis, table tennis, athletics, rowing, canoeing, cycling, cross-country skiing, alpine skiing, judo, triathlon, karate, trail running, paddle, badminton, orienteering, bicycle motocross, swimming, rhythmic gymnastics, climbing, and taekwondo Team sports: volleyball, basketball, ice hockey, handball, soccer, and water polo	DMFT Score: Individual sports: 8.12 (SD 3.56) Team sports: 6.1 (SD 3.63)	Not reported	Not reported	Individual Sports: 50/74 (68.9%) Team Sports: 62/112 (55.4%)	Individual sports: 7/72 (10.8%) Team sports: 19/112 (17%)	Not reported
Opazo-Garcia et al. [35]	2021	Athletics Handball Baseball Bowling Mountain Road cycling Soccer Hockey Judo Karate Wrestling Basque Racquetball Rowing Softball Squash Surf Taekwondo Tennis Triathlon Sailing Volleyball Beach	22/76 (29%)	7/76 (9%)	4/76 (5%)	Not reported	1% (1/76)	Not reported
Khan et al. [36]	2022	Athletics (track and field) Table tennis Lawn tennis Badminton Cricket Cycling Baseball Basketball Hockey	66/104 (63.5%) DMFT: 2.7 (SD 3.3)	27/104 (26.9%)	48/104(46.1%)	22/104 (21.15%)	Not reported	Not reported

SD = Standard Deviation; DMFT = Decayed, Missing, and Filled Teeth; GI = Gingival Index.

## Data Availability

The original contributions presented in this study are included in the Supplementary Materials. Further inquiries can be directed to the corresponding author.

## Supplementary Materials

The following supporting information can be downloaded at: https://www.mdpi.com/article/10.3390/dj13120589/s1, Supplementary Material File S1. PRISMA checklist; Table S1: Search Strategies; Table S2: Excluded articles; Table S3: JBI critical appraisal tool for included articles.

## Abbreviations

The following abbreviations are used in this manuscript:

CI	Confidence Interval
CINAHL	Cumulative Index to Nursing and Allied Health Literature
CoCoPop	Condition, Context, and Population
DMFT	Decayed, Missing, and Filled Teeth
GI	Gingival Index
JBI	Joanna Briggs Institute
LILACS	Latin American and Caribbean Health Sciences Literature
MOOSE	Meta-analysis Of Observational Studies in Epidemiology
PRISMA	Preferred Reporting Items for Systematic Reviews and Meta-Analyses
PROSPERO	International Prospective Register of Systematic Reviews
SD	Standard Deviation
WHO	World Health Organization
