# Supplementary material for: Prevalence and Severity of Oral Conditions in Elite Athletes: A Systematic Review and Meta-Analysis
Source: Dent J (Basel). 2025 Dec 8;13(12):589. doi: 10.3390/dj13120589 (PMC12731738; doi:10.3390/dj13120589)
Supplement: Supplementary file 1 [file dentistry-13-00589-s001.zip › Table S1_S2_S3.pdf]

Supplementary material

Supplementary Material Table S1. Search Strategies

**Database**	**PubMed**<br><br>**Date: 5/1/2025**		**Results**
**Search Strategy**	#1	Oral Health[Mesh]	22.100
	#2	Oral Health*[tiab]	40.757
	#3	Periodontal Diseases[Mesh]	99.811
	#4	Periodontal*[tiab]	77.414
	#5	Parodontos*[tiab]	209
	#6	Tooth[tiab]	115.631
	#7	Teeth[tiab]	143.915
	#8	Caries[tiab]	54.995
	#9	Carious[tiab]	8.722
	#10	Dental[tiab]	292.401
	#11	Denture*[tiab]	28.394
	#12	Dentin*[tiab]	39.342
	#13	Malocclusion*[tiab]	15.950
	#14	Periodonto*[tiab]	6.330
	#15	Gingiv*[tiab]	65.248
	#16	Pulpitis[tiab]	2.655
	#17	Endodontic*[tiab]	27.009
	#18	Stomatit*[tiab]	17.386
	#19	Temporomandibular[tiab]	26.487
	#20	Mandibular*[tiab]	81.784
	#21	Maxillofacial*[tiab]	27.813
	#22	Orofacial[tiab]	13.412
	#23	#1 OR #2 OR #3 OR #4 OR #5 OR #6 OR #7 OR #8 OR #9 OR #10 OR #11 OR #12 OR #13 OR #14 OR #15 OR #16 OR #17 OR #18 OR #19 OR #20 OR #21 OR #22	690.085
	#24	Athletes[Mesh]	23.363
	#25	Athlete*[tiab]	75.768
	#26	Athletic*[tiab]	25.322
	#27	Sportsman[tiab]	344
	#28	Sportsmen[tiab]	1.602
	#29	Sports[Mesh]	228.996

	#30	#24 OR #25 OR #26 OR #27 OR #28 OR #29	277.828
	#31	#23 AND #30	1.587

**Database**	**EMBase (Ovid)**<br><br>**Date: 5/1/2025**		**Results**
**Search Strategy**	#1	exp dental health/	5.742
	#2	(Oral adj3 Health*).ti,ab.	46.296
	#3	exp periodontal disease/	125.201
	#4	Periodontal*.ti,ab.	75.716
	#5	Parodontos*.ti,ab.	176
	#6	Tooth.ti,ab.	113.922
	#7	Teeth.ti,ab.	133.607
	#8	Caries.ti,ab.	49.861
	#9	Carious.ti,ab.	8.312
	#10	Dental.ti,ab.	268.748
	#11	Denture*.ti,ab.	25.528
	#12	Dentin*.ti,ab.	36.613
	#13	Malocclusion*.ti,ab.	14.460
	#14	Periodonto*.ti,ab.	5.847
	#15	Gingiv*.ti,ab.	65.412
	#16	Pulpitis.ti,ab.	2.246
	#17	Endodontic*.ti,ab.	24.059
	#18	Stomatit*.ti,ab.	20.328
	#19	Temporomandibular.ti,ab.	26.223
	#20	Mandibular*.ti,ab.	82.802
	#21	Maxillofacial*.ti,ab.	36.158
	#22	Orofacial.ti,ab.	14.342
	#23	or/1-22	675.630
	#24	exp athlete/	85.583
	#25	Athlete*.ti,ab.	87.277
	#26	Athletic*.ti,ab.	26.346
	#27	Sportsman.ti,ab.	480

	#28	Sportsmen.ti,ab.	2.310
	#29	exp sport/	230.601
	#30	or/24-29	302.021
	#31	23 and 30	2.079

**Database**	**CINAHL (EBSCO)**<br><br>**Date: 5/1/2025**		**Results**
**Search Strategy**	#1	(MM "Oral Health")	11.697
	#2	TI (Oral N3 Health*) OR AB (Oral N3 Health*)	20.696
	#3	(MH "Periodontal Diseases+")	24.202
	#4	TI Periodontal* OR AB Periodontal*	16.991
	#5	TI Parodontos* OR AB Parodontos*	0
	#6	TI Tooth OR AB Tooth	38.368
	#7	TI Teeth OR AB Teeth	38.368
	#8	TI Caries OR AB Caries	12.992
	#9	TI Carious OR AB Carious	1.706
	#10	TI Dental OR AB Dental	67.157
	#11	TI Denture* OR AB Denture*	4.925
	#12	TI Dentin* OR AB Dentin*	5.225
	#13	TI Malocclusion* OR AB Malocclusion*	2.889
	#14	TI Periodonto* OR AB Periodonto*	1.329
	#15	TI Gingiv* OR AB Gingiv*	12.456
	#16	TI Pulpitis OR AB Pulpitis	400
	#17	TI Endodontic* OR AB Endodontic*	4.047
	#18	TI Stomatit* OR AB Stomatit*	1.842
	#19	TI Temporomandibular OR AB Temporomandibular	6.883
	#20	TI Mandibular* OR AB Mandibular*	14.259
	#21	TI Maxillofacial* OR AB Maxillofacial*	5.806
	#22	TI Orofacial OR AB Orofacial	3.420
	#23	S1 OR S2 OR S3 OR S4 OR S5 OR S6 OR S7 OR S8 OR S9 OR S10 OR S11 OR S12 OR S13 OR S14 OR S15 OR S16 OR S17 OR S18 OR S19 OR S20 OR S21 OR S22	150.139

	#24	(MH "Athletes+")	38.175
	#25	TI Athlete* OR AB Athlete*	39.251
	#26	TI Athletic* OR AB Athletic*	13.004
	#27	TI Sportsman OR AB Sportsman	72
	#28	TI Sportsmen OR AB Sportsmen	230
	#29	(MH "Sports+")	95.041
	#30	S24 OR S25 OR S26 OR S27 OR S28 OR S29	120.616
	#31	S23 AND S30	563

**Database**	**Web of Science**<br><br>**Date: 5/1/2025**		**Results**
**Search Strategy**	#1	(TS=Oral Health OR TI=(Oral NEAR/3 Health*) OR AB=(Oral NEAR/3 Health*) OR TS=Periodontal Diseases OR TI=Periodontal* OR AB=Periodontal* OR TI=Parodontos* OR AB=Parodontos* OR TI=Tooth OR AB=Tooth OR TI=Teeth OR AB=Teeth OR TI=Caries OR AB=Caries OR TI=Carious OR AB=Carious OR TI=Dental OR AB=Dental OR TI=Denture* OR AB=Denture* OR TI=Dentin* OR AB=Dentin* OR TI=Malocclusion* OR AB=Malocclusion* OR TI=Periodonto* OR AB=Periodonto* OR TI=Gingiv* OR AB=Gingiv* OR TI=Pulpitis OR AB=Pulpitis OR TI=Endodontic* OR AB=Endodontic* OR TI=Stomatit* OR AB=Stomatit* OR TI=Temporomandibular OR AB=Temporomandibular OR TI=Mandibular* OR AB=Mandibular* OR TI=Maxillofacial* OR AB=Maxillofacial* OR TI=Orofacial OR AB=Orofacial) AND (TS=Athletes OR TI=Athlete* OR AB=Athlete* OR TI=Athletic* OR AB=Athletic* OR TI=Sportsman OR AB=Sportsman OR TI=Sportsmen OR AB=Sportsmen OR TS=Sports)	2.072

**Database**	**LILACS (BVS – Eng)**<br><br>**Date: 5/1/2025**		**Results**
**Search Strategy**	#1	((mh:(oral health) OR (oral) OR (bucal) OR (buccal) OR mh:(periodontal diseases) OR (periodontal*) OR (parodont*) OR (tooth) OR (diente*) OR (dente*) OR (teeth) OR (caries) OR (carious) OR (dental) OR (denture*) OR (dentin*) OR (malocclusion*) OR (maloclusión*) OR (ma-oclus*) OR (periodonto*) OR (gingiv*) OR (pulpitis) OR (endodont*) OR (stomatit*) OR (estomatit*) OR (temporomandibular) OR (mandibular*) OR (maxilofacial*) OR (orofacial*)) AND (mh:(athletes) OR (athlete*) OR (atleta*) OR (athletic*) OR (atleti*) OR (sportsman) OR (deportista*) OR (desportista*) OR (sportsmen) OR mh:(sports))) AND db:("LILACS") AND instance:"lilacsplus"	268

Database	Scopus<br><br>Date: 5/1/2025		Results
Search Strategy	#1	(INDEXTERMS ("Oral Health") OR TITLE-ABS ("Oral Health*") OR INDEXTERMS ("Periodontal Diseases") OR TITLE-ABS (periodontal*) OR TITLE-ABS (parodontos*) OR TITLE-ABS (tooth) OR TITLE-ABS (teeth) OR TITLE-ABS (caries) OR TITLE-ABS (carious) OR TITLE-ABS (dental) OR TITLE-ABS (denture*) OR TITLE-ABS (dentin*) OR TITLE-ABS (malocclusion*) OR TITLE-ABS (periodonto*) OR TITLE-ABS (gingiv*) OR TITLE-ABS (pulpitis) OR TITLE-ABS (endodontic*) OR TITLE-ABS (stomatit*) OR TITLE-ABS (temporomandibular) OR TITLE-ABS (mandibular*) OR TITLE-ABS (maxilofacial*) OR TITLE-ABS (orofacial)) AND (INDEXTERMS (athletes) OR TITLE-ABS (athlete*) OR TITLE-ABS (athletic*) OR TITLE-ABS (sportsman) OR TITLE-ABS (sportsmen) OR INDEXTERMS (sports))	1.631

Database	Dentistry & Oral Sciences Sources<br><br>Date: 5/1/2025		Results
Search Strategy	#1	((MH "Oral Health+") OR (TI "Oral Health*" OR AB "Oral Health*") OR (MH "Periodontal Diseases+") OR (TI Periodontal* OR AB Periodontal*) OR (TI Parodontos* OR AB Parodontos*) OR (TI Tooth OR AB Tooth) OR (TI Teeth OR AB Teeth) OR (TI Caries OR AB Caries) OR (TI Carious OR AB Carious) OR (TI Dental OR AB Dental) OR (TI Denture* OR AB Denture*) OR (TI Dentin* OR AB Dentin*) OR (TI Malocclusion* OR AB Malocclusion*) OR (TI Periodonto* OR AB Periodonto*) OR (TI Gingiv* OR AB Gingiv*) OR (TI Pulpitis OR AB Pulpitis) OR (TI Endodontic* OR AB Endodontic*) OR (TI Stomatit* OR AB Stomatit*) OR (TI Temporomandibular OR AB Temporomandibular) OR (TI Mandibular* OR AB Mandibular*) OR (TI Maxilofacial* OR AB Maxilofacial*) OR (TI Orofacial OR AB Orofacial)) AND ((MH Athletes+) OR (TI Athlete* OR AB Athlete*) OR (TI Athletic* OR AB Athletic*) OR (TI Sportsman OR AB Sportsman) OR (TI Sportsmen OR AB Sportsmen) OR (MH Sports+))	359

Supplementary Material Table S2. Excluded articles

Author	Year of publication	Reason of exclusion
Dental Nursing	2015	No outcomes of interest
Alajbeg et al	2017	No outcomes of interest
Bemelmanns et al	2000	Published in German
Botelho et al	2021	Did not include the target population (elite athletes)
Bryant et al	2011	Did not include the target population (elite athletes)
Chantaramanee et al	2016	Did not include the target population (elite athletes)
Chapman et al	1985	Did not include the target population (elite athletes)
Hajiyev et al	2022	Did not include the target population (elite athletes)
Hajiyev et al	2020	No outcomes of interest
Minty et al	2018	No outcomes of interest
Randell et al	1983	No outcomes of interest
Amy et al	2005	No outcomes of interest
Bawazir et al	2022	No outcomes of interest
Bergman et al	2017	No outcomes of interest
Chapman et al	2013	No outcomes of interest
Hendrick et al	2008	No outcomes of interest
Keçeci, Ayşe et al	2005	No outcomes of interest
Padilha et al	2020	No outcomes of interest

Supplementary Material Table S3. JBI critical appraisal tool for included articles.

	**Was the sample frame appropriate to address the target population?**	**Were study participants sampled in an appropriate way?**	**Was the sample size adequate?**	**Were the study subjects and the setting described in detail?**	**Was the data analysis conducted with sufficient coverage of the identified sample?**	**Were valid methods used for the identification of the condition?**	**Was the condition measured in a standard, reliable way for all participants?**	**Was there appropriate statistical analysis?**	**Was the response rate adequate, and if not, was the low response rate managed appropriately?**
Khan et al	Yes	No	No	Yes	Yes	Yes	Yes	Yes	Yes
Opazo-García et al.	Yes	No	No	Yes	Yes	Yes	Yes	Yes	Unclear
Andrade et al	Yes	No	No	Yes	Yes	Yes	Yes	Yes	Yes
Gay Escoda et al.	Yes	No	No	No	Yes	Yes	Yes	Yes	Yes
Needleman et al	Yes	No	No	Yes	Yes	Yes	Yes	Yes	Yes
Needleman et al	Yes	No	No	Yes	Yes	Yes	Yes	Yes	Yes
Gallagher et al	Yes	No	No	Yes	Yes	Yes	Yes	Yes	Yes
Kragt et al	Yes	No	No	Yes	Yes	Yes	Yes	Yes	Yes
De la Parte et al.	Yes	No	No	Yes	Yes	Yes	Yes	Yes	Yes
Cardoso et al	Yes	No	No	Yes	Yes	Yes	Yes	Yes	Yes
